# Molecular-Based Detection of *Leishmania infantum* in Human Blood Samples in a New Focus of Visceral Leishmaniasis in Lorestan Province, Iran

**Published:** 2018-03-18

**Authors:** Leila Masoori, Farnaz Kheirandish, Ali Haghighi, Mehdi Mohebali, Behnaz Akhoundi, Niloofar Taghipour, Latif Gachkar, Ali Chegeni-Sharafi, Vahideh Moin-Vaziri

**Affiliations:** 1 Department of Parasitology and Mycology, School of Medicine, Shahid Beheshti University of Medical Sciences, Tehran, Iran; 2 Department of Parasitology and Mycology, School of Medicine, Lorestan University of Medical Sciences, Khorramabad, Iran; 3 Department of Medical Parasitology and Mycology, School of Public Health, Tehran University of Medical Sciences, Tehran, Iran; 4 Center for Research of Endemic Parasites of Iran (CREPI), Tehran University of Medical Sciences, Tehran, Iran; 5 Infectious Diseases and Tropical Medicine Research Center, Shahid Beheshti University of Medical Sciences, Tehran, Iran; 6 Department of Communication Disease Control and Prevention, Deputy of Health, Lorestan University of Medical Sciences, Khorramabad, Iran

**Keywords:** Visceral leishmaniasis, *Leishmania infantum*, kDNA, ITS1, Iran

## Abstract

**Background:**

The fatal form of leishmaniasis is visceral form (VL), found in some of the countries in the world. Visceral leishmaniasis has been reported sporadically from all provinces in Iran, including Lorestan. This study aimed to characterize parasite species in DAT positive and some of the DAT negative human blood samples of Delfan district, Lorestan Province, central Iran.

**Methods:**

Blood **s**amples were collected from different geographical areas of Delfan. Serum was used for DAT test and remained part of molecular study. DNA was extracted by using DNG-plus extracted kit (Cinagen, Iran). Polymerase chain reaction amplification of *Leishmania* kDNA and PCR-RFLP of ITS1 was done to identify *Leishmania* species. Some amplicons were sequenced, submitted to GenBank and analyzed by BLASTn.

**Results:**

Expected band of kDNA for *L. infantum* (720bp) was amplified in 16 out of 186 (8.6%) samples which showed previously anti-*Leishmania* antibody at different titers or were negative serologically. Using BLASTn, 93% similarity with *L. infantum* has been shown. The rDNA-ITS1 was amplified only in 9 samples (4.7%). RFLP pattern was similar to what expected for *L. infantum*.

**Conclusion:**

A new emerging hypo-endemic focus, caused by *L. infantum*, is going to be established in Delphan District, Lorestan Province. Further studies on vector and reservoirs are necessary for the region and other parts of Lorestan Province.

## Introduction

Leishmaniasis is a group of protozoan diseases transmitted to humans and other mammals by Phlebotominae sandflies and can manifest in different clinical forms, depending upon the infecting species of *Leishmania*. The disease could emerge as cutaneous, mucocutaneous or Visceral Leishmaniasis (VL) (1). Mediterranean form of VL is defined as a zoonotic disease, VL is a huge burden on human health and society, it is caused by *L. donovani* complex in Asia and Africa and by *L. chagasi*, a synonymous of *L. infantum*, in Latin America (2–4).

The leishmaniasis is found to be prevalent in some of the poorest countries in the world, and they gain lesser attention than another infectious disease like malaria, tuberculosis, and AIDS. They are categorized as a neglected tropical disease (NTD) because although not recognized and prioritized, they cause significant health problems to the very poorest in society. There is little effort on the part of the global community and pharmaceutical industry to invest in research and development of better and innovative therapeutic because of lack of sufficient incentives (1, 5).

Leishmaniasis rank as the leading NTD in terms of mortality, with an estimated 20000–40000 death (6, 7) and 3.3 million disability-adjusted life years (8).

The most life-threating form of disease is VL, in which the pathogen disseminates to endothelial systems, like liver, spleen and bone marrow. Clinical signs and symptoms of VL generally include prolonged and irregular fever associated with chills, hepatosplenomegaly, lymphadenopathy, progressive anemia, weight loss. More than 90% of VL cases are reported from Bangladesh, Brazil, Ethiopia, India, Sudan and South Sudan (8).

Two different forms of leishmaniasis comprising VL and CL exist in Iran. Cases of VL have been reported from all parts of the country (9). Unlike CL, which accounts for almost 20000 new cases per year (9), VL has been reported sporadically, with endemic foci located in northwestern and southern Iran, with about 100–300 new cases annually (9, 10).

The diagnosis of VL is a challenging issue and several studies work to resolve problem (4). Direct agglutination test (DAT) has been applied vastly for serological studies of VL in human and animal reservoir hosts in the world as well in Iran particularly in the endemic areas (9, 11–13). DAT is a simple, cost-effective and field applicable test and has been recommended for seroepidemiological studies as well as early and accurate diagnosis of VL, especially in endemic areas (9, 14).

As mentioned VL is reported sporadically in all provinces in Iran, there are several reports of sporadic VL cases in Lorestan Province, but real status of disease is not clear in this area. Lorestan is a province of western Iran in the Zagros Mountains, the climate is generally sub-humid continental with cold winter. A seroepidemiological study using DAT was performed on 800 collected sera from healthy population of Delphan County located in mountainous part of Lorestan Province by the same authors. Anti-*Leishmania* antibody at different titers in 38 cases (4.75%) which indicate that a new focus of VL with low endemicity is going to be formed in Delphan district (15). Therefore, further studies seem necessary on *Leishmania* species identification, vector incrimination, as well as reservoirs.

Identification of *Leishmania* parasites is essential for precise prognosis of the disease as well as making proper decision regarding treatment (16, 17). DNA-based methods have proven to be effective in detecting the genome of *Leishmania* species in different biological samples with aim of species identification (4).

Among different genetic markers used for *Leishmania* identification, kDNA, and ITS1 vastly used in detecting parasite in different biological samples (18–20). The kinetoplast DNA contains around 10000 mini-circles per cell, each around 800bp in length with an approximately 600bp variable and 200bp conserved region. The heterogeneity of the variable region has been exploited to discriminate between *Leishmania* spp (21). ITS1-RFLP-PCR also is common in characterization of different *Leishmania* spp (16, 20, 22, 23).

The current study aimed to characterize *Leishmania* species in the peripheral blood samples which showed anti-*Leishmania* antibodies at titer ≥1:3200 (considered positive) and titers between 1:800 and 1:1600 (considered suspected) and 20% of titers under 1:800 (considered negative) obtained from previous study (15), targeting kDNA and ITS1 gene.

## Materials and Methods

### Study area

Delfan district is located in mountainous area of northwest of Lorestan Province. The altitude of study area is about 2000m above sea level and very cold in winter. The total population was 85000 in 2009, including 1200 nomadic family.

### Sample preparation

Totally 800 samples were collected from children ≤ 12yr old and 10% of adults by a multi-stage randomized cluster sampling in 2012. After filling out a questionnaire, 3ml of peripheral blood sample has been taken from each person. Sera of about 1ml of blood sample had been used for serology study, using DAT at the School of Public Health, Tehran University of Medical Sciences, Tehran, Iran (10) and results published previously (15). Two remained ml were kept at −20 °C in the EDTA-containing tubes in Department of Parasitology, Shahid Beheshti University of Medical Sciences, Tehran, Iran for DNA extraction.

Based on serology results (15), just 186 samples out of 800 which showed anti-*Leishmania* antibody at different titers were subjected to molecular works, comprising 5 cases (0.62%) at titers ≥ 1:3200 considered as positive, 21 samples (2.62%) at titer 1:800 to 1:1600 considered suspected and 160 samples (20%) serologically negative (out of them 146 showed no titer and 14 at titer 1:400) (Table 1).

**Table 1.** Number of molecular positive samples at different anti-*Leishmania* antibody titer among collected human blood samples from Delfan District, Lorstan Province, Iran, 2012

**Antibody Titer**		**Number of samples**	**Molecular positive cases**	**Percent**
**Negative**	**No titer**	146	1	0.68
	**1:400**	14	5	35.7

**Suspected: (1:800 to 1:1600)**		21	9	42.8
**Positive: (≥ 1: 3200)**		5	1	20
**Total**		186	16	8.6

### DNA Extraction

Five hundred microliters of whole blood were used for DNA extraction. To remove interfering hemoglobin molecules from the samples prior to DNA extraction, blood was washed with PBS, even 10 times. DNA was extracted using DNG-plus extracted kit (Cinagen, Iran) based on manufacturer instructions, DNA concentration was determined by NanoDrop (Bio wave II) at 220 and 280nm. Standard strains *L. infantum*: MCAN/IR/07/Moheb.gh, *L. tropica*: MHOM/IR/02/Mash10 and *L. major*: MRHO/IR/75/ER was achieved from Department of Medical Parasitology, School of Public Health, Tehran University of Medical Sciences, Iran to monitor the reactions.

### PCR amplification of *Leishmania* kDNA

For initial parasite detection, a nested-PCR was performed by using specific primers CSB2XF: (CGAGTAGCAGAAACTCCCG TTCA), CSB1XR: (ATTTTTCGCGATTTT CGCAGAACG) (external) and 13Z: (ACT GGGGGTTGGTGTAAAATAG), LiR: (TC GCAGAACGCCCCT) (internal) (21). These primers amplified a fragment of 560 to 750 bp species dependent. The amplification conditions of both two rounds were 94 °C for 5 min, followed by 30 cycles of denaturation at 94 °C for 60 sec, annealing at 55 °C for 60 sec and extension at 72 °C for 90sec, with a final extension step at 72 °C for 5min. Five μl of the PCR production of second rounds were visualized on a UV transilluminator following 1.5% agarose gel electrophoresis containing ethidium bromide.

### PCR amplification for *Leishmania* ITS1

LITSR and L5.8 primers were used to amplify the small subunit 5.8s rRNA of parasite genome. Specific primers, LITSR (forward: 5′-CTGGATCATTTTCCGATG-3′) and L5.8S: (reverse: 5′-TGATACCACTTATCGCACTT-3′), then PCR products were digested by HaeIII (BsuRI) restriction enzyme following protocol (24). After using the restriction enzyme, pattern fragment of 200, 80 and 60bp for *L. infantum* would be produced and visualized by 2% agarose gel electrophoresis, stained with ethidium bromide. All the procedures were monitored by standard *Leishmania* parasite (*L. major* Acc. No: JN860745, *L. tropica* Acc. No: EF653267 and *L. infantum* (Acc. No: FJ555210).

### Sequencing

Some samples were sequenced, submitted to GenBank and their homology with the available sequence data in GenBank was checked by using BLASTn (25).

### Ethics

Ethics clearance was obtained from Research Ethical Community of Shahid Beheshti University of Medical Sciences (Approval Number: SMBU.REC.1392.302).

## Results

### *Leishmania* parasite identification by kDNA-Nested-PCR

**Fig. 1.**
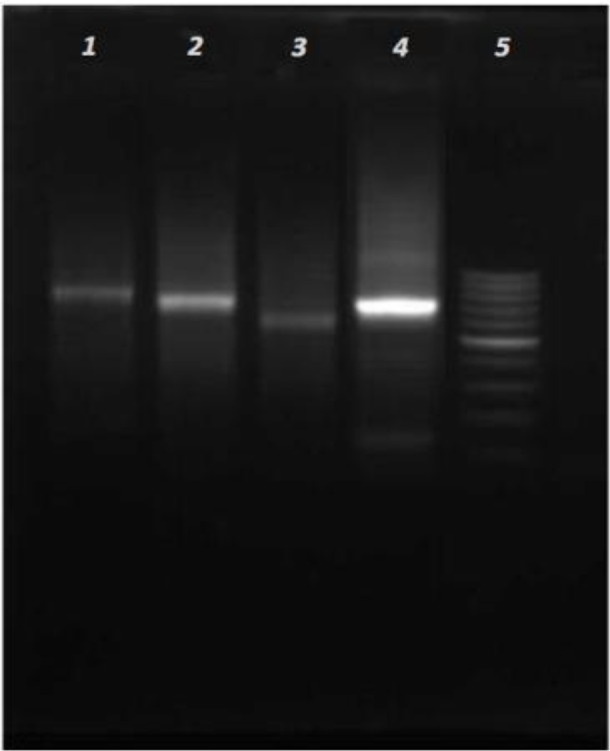
Gel electrophoresis of kDNA-Nested-PCR products obtained from *L. infantum* isolates of blood samples collected from Delfan district, Lorestan Province

In 16 samples out of 186 ones (8.6%), amplification of kDNA was yielded by visualizing band of 720bp, for *L. infantum* (Fig. 1). The kDNA amplification was obtained from 6 serologically negative samples, including one sample with no titer and 5 others at titer 1:400 (Table 1).

The PCR products of 6 samples were sequenced and deposited in GenBank (Accession no: KJ417490, KJ417491, KJ417492, KJ417493, KJ417494, KJ417495). BLAST analysis showed 83–93% homology to *L. donovani* (Accession no: AJ100780.1) and *L. infantum* (Accession no.NZ32847.1).

### *Leishmania* parasite identification with ITS1-PCR-RFLP

**Fig. 2.**
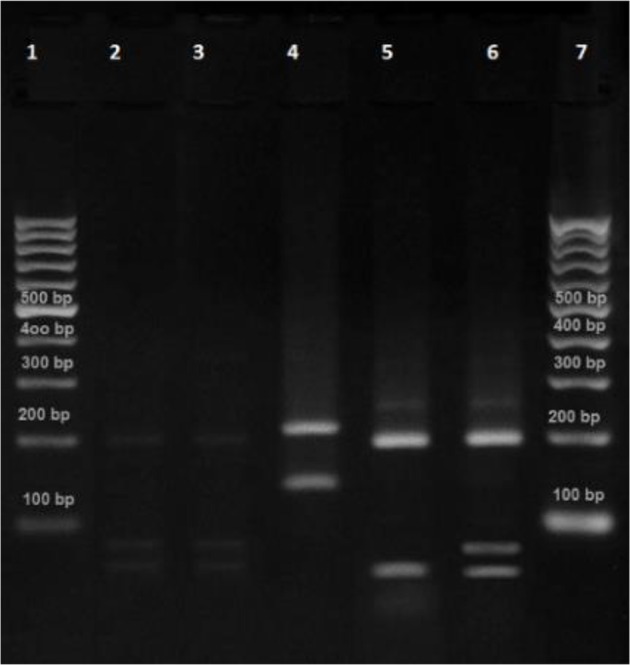
Digestion of ITS1-rDNA amplicons using *HaeIII* (*BsuRI*) enzyme of *Leishmania* isolated from blood samples collected from Delfan district, Lorestan Province

Totally 9 samples (including sero-negative cases) out of 189 (4.7%) have shown *Leishmania* infection by visualizing a band approximately 350bp in 1.5% agarose gel. Digestion of amplicons with HaeIII (BsuRI) enzyme produced the fragments of 200, 80 and 60bp characterized as *L. infantum* in all samples. The results also were monitored by digestion pattern in reference strain of *L. infantum* (Fig. 2).

One PCR product was sequenced and submitted to GenBank, which is accessible by Accession no. KJ417496. BLAST analysis showed 94% similarity to Iranian *L. infantum*. (Accession no: KC34730 and HQ535858.1).

Lane 1–3: *Leishmania tropica* MHOM/IR/02/Mash10 (750bp), *L. infantum* MCAN/IR/07/Moheb.gh (720bp), L. *major* MHOM/IR/75/ER (560bp), standard strains respectively

Lane 4: Sample of current study (*L. infantum*)

Lane 5: Molecular weight marker (100bp ladder Fermentas)

Lane 1 and 7: M: Molecular weight marker (100 bp ladder Fermentas)

Lane 2 and 3: Current study samples

Lane 4*–*6: *Leishmania tropica* MHOM/IR/02/Mash10, *L. infantum* MCAN/IR/07/Moheb.gh, *L. major* MHOM/IR/75/ER standard strains respectively

## Discussion

VL is a life threating disease with an estimation of 50000 deaths in 2010 in the world (6). VL occurs sporadically in all geographical zones of Iran but it is endemic in some areas mainly located in northwestern and southern part of the country. Disease status in Lorestan Province is not clearly distinct. Our previous study in Delfan District (located in northwest of Lorestan Province) showed 3.25% sero-positive samples among 800 collected ones using DAT (15). *Leishmania* species identification is of great importance from clinical and epidemiological point of view. As a result of morphological similarities, *Leishmania* species could not be firmly identified using conventional methods. Main objective of current study was to characterize the *Leishmania* species among the samples which collected from Delfan and as a result of our previous study showed anti-*Leishmania* antibodies at different titers (15), targeting two different molecular markers.

kDNA is introduced as a quite useful marker for initial screening of parasite by different researchers (26, 27). In the present study, kDNA could be a reliable marker because peripheral blood samples were used. A fragment about 720bp of kDNA was visualized in 16 out of 186 samples (8.6%), which based on the size and sequencing results they were characterized as *L. infantum*. This marker is used commonly in distinguishing parasite in human samples, reservoirs, and vectors, most especially when there was not enough amount of parasite in the samples (21, 23, 28, 29). Using this marker, *L. infantum* was isolated from sandflies in Greece by producing a fragment of 720bp of parasite genome (30). *Leishmania infantum* infection was identified in asymptomatic dogs in a new endemic focus of VL in Iran (31).

Another useful marker which vastly used for *Leishmania* species identification is ITS1 (24, 32–37). In the current study, expected band of 350bp length was amplified just in 9 out of 186 samples (4.8%). Compared to kDNA positive cases (8.4%) it could be concluded that kDNA is quite appropriate for initial screening especially in case of inadequate amount of parasite in clinical samples. This marker could detect 0.01 to 0.001 parasites per ml whereas ITS1 has ability of detection of 1–6 parasites per ml (37, 38). Enzymatic digestion pattern of ITS1 amplicons was the same as what expected for *L. infantum* which verified the results obtained using kDNA.

Both genetic markers could detect parasite in one sample considered serologically negative. It was a recent infection which emphasizes on the ability of molecular tools in early detection of parasite, especially reasoning the ability of kDNA marker in initial screening of the samples. If we had chance to follow the case, it would have shown rising titer, unfortunately, it was not possible for authors in spite of several attempts. DNA of *Leishmania* parasite also was detected in 5 samples out of 14 (35.7%) which showed anti-*Leishmania* antibodies at titer 1/400, which according to the interpretation of DAT results, they were considered negative. In these cases also rising titer was probable, if follow up was possible.

## Conclusion

Altogether, seroepidemiology and molecular results of current survey showed that a new emerging hypo-endemic focus is going to be established in Delfan District, Lorestan Province, due to *L. infantum*. Considering the lifestyle, climate and massive migration for seeking job, necessity of further work on other epidemiological aspects of disease are evident, as well health education should be considered by health authorities.
